# Prognosis and Early Diagnosis of Ductal and Lobular Type in Breast Cancer Patient

**Published:** 2017-11

**Authors:** Houriyeh EHTEMAM, Mitra MONTAZERI, Reza KHAJOUEI, Raziyeh HOSSEINI, Ali NEMATI, Vahid MAAZED

**Affiliations:** 1 Health Services Management Research Center, Institute for Futures Studies in Health, Kerman University of Medical Sciences, Kerman, Iran; 2 Modeling in Health Research Center, Institute for Futures Studies in Health, Kerman University of Medical Sciences, Kerman, Iran; 3 Computer Engineering Department, Faculty of Engineering, Shahid Bahonar University, Kerman, Iran; 4 Medical Informatics Research Center, Institute for Futures Studies in Health, Kerman University of Medical Sciences, Kerman, Iran; 5 Health Information Sciences Dept., Faculty of Management and Medical Information Sciences, Kerman University of Medical Sciences, Kerman, Iran; 6 Hematology and Oncology, Faculty of Medicine, Kerman University of Medical Sciences, Kerman, Iran

**Keywords:** Diagnosis, Breast cancer, Ductal and lobular, Data mining models

## Abstract

**Background:**

Breast cancer is one of the most common cancers with a high mortality rate among women. Prognosis and early diagnosis of breast cancer among women society reduce considerable rate of their mortality. Nowadays, due to this illness, try to be setting up intelligent systems, which can predict and early diagnose this cancer, and reduce mortality of women society.

**Methods:**

Overall, 208 samples were collected from 2014 to 2015 from two oncologist offices and Javadalaemeh Clinic in Kerman, southeastern Iran. Data source was medical records of patients, then 64 data mining models in MATLAB and WEKA software were used, eventually these measured precision and accuracy of data mining models.

**Results:**

Among 64 data mining models, Bayes-Net model had 95.67% of accuracy and 95.70% of precision; therefore, was introduced as the best model for prognosis and diagnosis of breast cancer.

**Conclusion:**

Intelligent and reliable data mining models are proposed. Hence, these models are recommended as a useful tool for breast cancer prediction as well as medical decision-making.

## Introduction

Cancer leads to physical and emotional stress (1) among all kinds of cancers is the most common cancer (2). Moreover, it has ascending growth in deprived areas (3). Surprisingly, this illness is rare among men. However, it is the most common cause of death in women (2). Breast cancer has various morphologies, which are used in classifying of this disease (4). Some researchers consider Ductal and Lobular to classify types of this cancer. These two morphologies (Ductal and Lobular) have different characteristics, but Ductal is the most common type, and approximately it has allocated 75% to 85% of breast cancers to own (5).

Identifying risk factors of breast cancer has become an important issue among physicians and pathologists (6). However, by medical technologies improvements, useful risk factors are measuring and recording (7). Early diagnosis of breast cancer is very effective in re-cover of patients, and it has positive impact on longevity of them. In spite of this cancer is so common, it will be the most curable when detect soon (8). Early diagnosis of breast cancer is very effective in recovery of the disease, and it has positive impact on longevity of patients, although this cancer is the most common types of cancer among women, it will be the most curable when detected early (9). In order to diagnosis of breast cancer, intelligent models are useful to increase the precision and accuracy of diagnosis (10). By advancement in computerized software and hardware, the massive volume of data is recorded automatically, after that efficient analysis methods help to analyze the data efficiently (7).

Data mining is one of the technology improvements that serve to manage data. Widespread use of information systems lead to merge data mining with traditional methods (11).

Utilization of data mining techniques with the approach of extracting knowledge from information have many advantages, such as how to recognize diseases, reducing health care costs, reducing medical errors, and last but not least improve the performance of healthcare organizations (12).

**Fig. 1:**
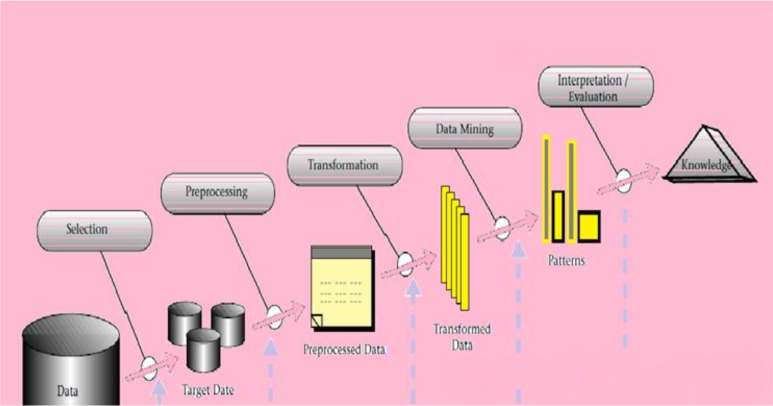
Steps of knowledge discovery in databases with data mining process (16)

Additionally, data-mining models can be a way to reduce errors in decision making by physicians. In medical levels, data mining effort is used to extract relationships and patterns from a large number of data to predict diseases (13). The result of these analyses should be comprehensible for everyone (14). Totally, data mining is defined as a process of selecting, exploring and modeling large volume of data used in order to discover new and usable patterns from data analyzing (15). According to Fig. 1, steps of extracting knowledge from database by using data mining were depicted in five stages.

In the first stage, special data was selected among large volume of data. In the second stage, preprocessing methods was performed on data, for instance controlling a missing data. In third step, data were ready to transform based on hypothesis. Then, data-mining algorithms were selected, they decide about which patterns are more appropriate. In fifth stage, interpretation/evaluation was done. All previous steps will be evaluated again. Consequently, it prepared us an image from extracted patterns and models. Knowledge was the final product of this process. Eventually, we could present this knowledge without combined to other systems, or report it to other enthusiastic people (16).

Hence, we can use this intelligent method as accurate and reliable system to early diagnosis of benign or malignant of breast cancer (17). This method could lead to save many people from threat of death due to breast cancer, or enhance their longevity and quality of their life.

In this study, we aimed to present the most effective data mining models to identify breast cancer sooner.

## Materials and Methods

### Data collection

A list of breast cancer risk factors was taken from a previous study (18), and then they were confirmed by an oncologist. Samples based on these risk factors were gathered from records of breast cancer patients, and whole of their identity information kept secret. Medical records of 208 patients collected from two oncologist offices, and Javadalaemeh Clinic, from 2014 to 2015. In order to control missing data, the most frequent repeat was replaced for discrete data, and for continuous missing one, the average of data in corresponding column is replaced (19).

These risk factors are as follow: age, sex, BMI, Marital Status (MS), Age Starting of First Menstruation (ASFM), the Number of Parturition (NP), the Number of Abortion (NA), Age Starting of Menopause (ASM), History of Breast Cancer, Uterine and Ovarian Cancer in First-Degree Relatives(HBCUOCFDR), History of Breast Cancer, Uterine and Ovarian Cancer in Second-Degree Relatives (HBCUOCSDR), History of Other Cancers in First-Degree Relatives (HOCFDR), History of Other Cancers in Second-Degree Relatives (HOCSDR), ER, PR, Existence of Tumor (ET), Size of Tumor (ST), Type of Cancer (TC).

### Risk factors

Overall, 17 risk factors for breast cancer were used. The risk factors were divided into two groups (nominal and real). These risk factors are as follow: age (yr), sex (Male/Female), BMI (kg/m^2^), Marital Status (Single/Married), Age Starting of First Menstruation (yr), the Number of Parturition (Number), the Number of Abortion (Number), Age Starting of Menopause (yr), History of Breast Cancer, Uterine and Ovarian Cancer in First-Degree Relatives(Yes/No), History of Breast Cancer, Uterine and Ovarian Cancer in Second-Degree Relatives (Yes/No), History of Other Cancers in First-Degree Relatives (Yes/No), History of Other Cancers in Second-Degree Relatives (Yes/No), ER (Positive/Negative), PR (Positive/Negative), Existence of Tumor (Yes/No), Size of Tumor (Cm), Type of Cancer (Ductal/Lobular).

### Classification

**Fig. 2:**
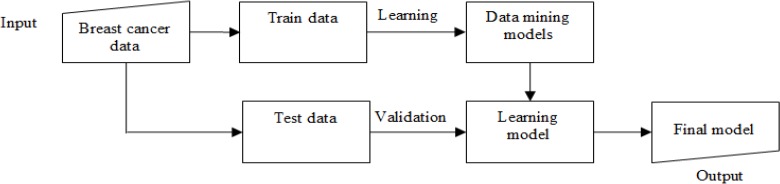
Flow chart of proposed method

The data were analyzed by WEKA and MATLAB software, and 64 data mining models classified them. Of all 17 risk factors, 16 of them were defined as independent risk factors, and one of them that was a specified type of cancer divided into Ductal and Lobular allocated class (dependent risk factor) tag to own. The stages of our method are shown in Fig. 2. Initially, the collected breast cancer data were considered as input. Secondly, the data divided into train and test kind. In third stage, train data were learned based on a special technique and produce data mining models. After that, the model changed to learned model. In fourth step, the performance of the learned model became valid by test data. Finally, the final model was presented as output.

### Experimental findings

#### Configuration of the proposed models

Samples that were belong to positive and negative class, were denoted as *P* and *N*, respectively. In each classification, four definitions can be explained as follow:

- positive group and anticipate correctly called True Positive (*TP*).
- positive group and anticipate incorrectly called False Positive (*FP*).
- negative group and anticipate correctly called True Negative (*TN*).
- negative group and anticipate incorrectly called False Negative (*FN*).

Therefore, the equations for precision and accuracy can be defined as follow:

(1) $$\text{Precision}=\frac{\text{TP}}{\left(\text{TP}+\text{FP}\right)}$$

(2) $$\text{Accuracy}=\frac{\left(\text{TP}+\text{TN}\right)}{\left(\text{P}+\text{N}\right)}$$

## Results

After choosing effective risk factors, two morphologies of this cancer were considered (Ductal and Lobular). Another phase of this paper was data mining. In this phase the data became valid by a special method explained in section entitle “method” and the valid data after some other process produced final model. In order to evaluation*, K*-Fold cross validation method was used. *K* was equal to 10 (*K*=10).

The results of the Binomial Test are shown in Table 1. First phase of our work was presented in Table 1 that was designed by SPSS software (Chicago, IL, USA). Error was reported as 0.05. In addition, the value of *P*-value for Ductal and Lobular had been achieved 0; thus, our final method had high accuracy.

**Table 1:** *P*-value which compares *P*-value between two type of breast cancer (Ductal and Lobular)

***Binomial Test Type of cancer***	***Category***	***n***	***Observed Prop.***	***Test Prop.***	***P-Value***
Group 1	Ductal	198	.95	.50	.000
Group 2	Lobular	10	.05		
Total		208	1.00		

Table 2 presents nominal risk factors (ER, PR, tumor size, parity, marital status and age) which were grouped based on frequency, percent, valid percent, and cumulative percent. In Table 3, 64 data mining models are shown. There are percentages of accuracy and precision, too.

**Table 2:** Grouping of nominal risk factors of breast cancer

***Risk factor***	***Group***	***Frequency***	***Percent^1^***	***Valid Percent^2^***	***Cumulative Percent^3^***
ER	Positive	137	65.2	65.2	66.2
	Negative	71	33.8	33.8	100.0
	Total	208	100.0	100.0	-
PR	Positive	148	70.5	70.5	71.4
	Negative	60	28.6	28.6	100.0
	Total	208	100.0	100.0	-
MS	Married	196	93.3	94.2	94.2
	Single	12	5.7	5.8	100.0
	Total	208	99.0	100.0	-
ET	Yes	179	86.1	86.1	86.1
	No	29	13.9	13.9	100.0
	Total	208	100.0	100.0	-
HBCUOCFDR	Yes	15	7.2	7.2	7.2
	No	193	92.8	92.8	100.0
	Total	208	100.0	100.0	-
HBCUOCSDR	Yes	17	8.2	8.2	8.2
	No	191	91.8	91.8	100.0
	Total	208	100.0	100.0	-
HOCFDR	Yes	19	9.1	9.1	9.1
	No	189	90.9	90.9	100.0
	Total	208	100.0	100.0	-
HOCSDR	Yes	18	8.7	8.7	8.7
	No	190	91.3	91.3	100.0
	Total	208	100.0	100.0	-
Parity	0–5	179	85.2	86.1	86.1
	6–11	27	12.9	13.0	99.0
	12–17	2	1.0	1.0	100.0
	Total	208	99.0	100.0	-
TC	Ductal	198	95.2	95.2	95.2
	Lobular	10	4.8	4.8	100.0
	Total	208	100.0	100.0	-

1 Represents the percentages of all data, including the missing data, established by each category.

2 Valid percent presents only the non-missing cases.

3 Cumulative percent brings an easier way to compare different sets of data.

**Table 3:** Amount of precision and accuracy of the each model

***NO.***	***Machine learning model***	***Classification accuracy (%)***	***Precision (%)***
1.	Bayes-Net (20)	95.67	95.70
2.	Naïve-Bayes	91.83	95.00
3.	Naïve-Bayes-Updateable	91.83	95.00
4.	Logistic	90.86	95.00
5.	Multilayer-Perceptron	91.83	95.50
6.	RBF-Network	94.23	95.10
7.	Simple-Logistic	95.19	95.20
8.	Sequential-Minimal Optimization (21)	95.19	95.20
9.	Voted-Perceptron	95.19	95.20
10.	Instance-Based-Learning-algorithms	90.86	95.00
11.	IBK	90.38	95.00
12.	K-Star	91.82	95.00
13.	Locally-Weighted-Learning	94.71	95.20
14.	AdaBoost-ML	95.19	95.20
15.	Attribute-Selected-Classifier (22)	95.19	95.20
16.	Bagging	95.19	95.20
17.	Classification-Via-Clustering	69.23	94.70
18.	Classification-Via-Regression	94.71	95.20
19.	Cross-Validation-Parameter-Selection (23)	95.19	95.20
20.	Dagging	95.19	95.20
21.	Decorate (24)	95.19	95.20
22.	Ensembles of Nested Dichotomies (25)	95.19	95.20
23.	Ensemble-Selection (26)	95.19	95.20
24.	Filtered-Classifier (22)	95.19	95.20
25.	Grading	95.19	95.20
26.	Logit-Boost	95.19	95.20
27.	Multi-Boost-AB (27)	95.19	95.20
28.	Multi-Class-Classifier	90.86	95.00
29.	Multi-Scheme	95.19	95.20
30.	Ordinal-Class-Classifier (28)	95.19	95.20
31.	Raced-Incremental-Logit-Boost (29)	95.19	95.20
32.	Random-Committee	94.23	95.10
33.	Random-Sub-Space (30)	95.19	95.20
34.	Rotation-Forest	95.19	95.20
35.	Stacking	95.19	95.20
36.	Stacking-C	95.19	95.20
37.	Threshold-Selector	94.23	95.10
38.	Vote	95.19	95.20
39.	Hyper-Pipes (31)	95.19	95.20
40.	classification by Voting Feature Intervals	74.52	95.60
41.	Conjunctive-Rule	95.19	95.20
42.	Decision-Table	95.19	95.20
43.	Decision-Table-Naïve-Bayes (32)	95.19	95.20
44.	J-Repeated-incremental-pruning (33)	95.19	95.20
45.	Non-Nested-generalized-exemplars	92.79	95.10
46.	One-R (34)	95.19	95.20
47.	PART	94.23	95.10
48.	Ridor(35)	95.19	95.20
49.	Zero-R	95.19	95.20
50.	Alternating-Decision Tree (36)	95.19	95.20
51.	Best-FirstTree	95.19	95.20
52.	Decision-Stump	95.19	95.20
53.	Functional trees	94.71	95.20
54.	J48 (37)	95.19	95.20
55.	J48-graft (38)	95.19	95.20
56.	LAD-Tree	91.35	95.20
57.	NB-Tree (39)	95.19	95.60
58.	Random-Forest	93.75	95.10
59.	Random-Tree	90.38	95.40
60.	REP-Tree (40)	95.19	95.20
61.	Simple-Cart	95.24	95.20
62.	Class-Balanced-Nested-Dichotomies (41)	95.19	95.20
63.	(Data-Near-Balanced-ND (41)	95.19	95.20
64.	Nested-Dichotomies	95.19	95.20

**Fig. 3:**
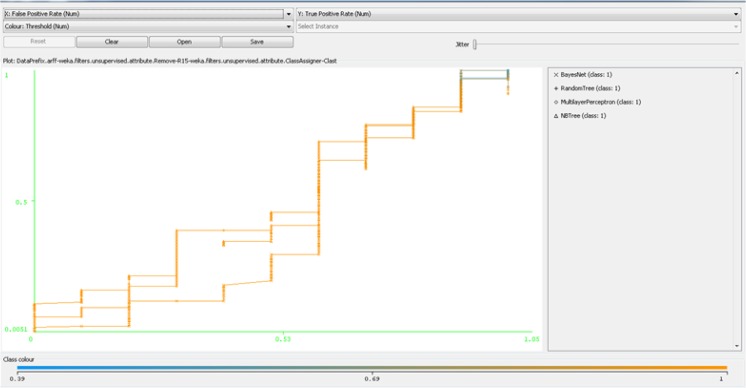
ROC curve of four best models in WEKA software (BN, MP, NB-Tree, and RT)

As it is obvious in Table 3, VFI were the weakest model in prognosis and diagnosis of breast cancer, and Bayes-Net was identified as the best. Fig. 3 demonstrates the ROC curve of the four best models among 64 models (BN, MP, NB-Tree, and RT). This figure shows the performance of these models in WEKA software. The MP model has the highest ROC area value among the other four models.

## Discussion

Breast cancer is one of the most common cancers in women. Early detection of breast cancer leads to declining mortality. Technology improvements can help early diagnosis of breast cancer. Data mining method is an intelligent model that diagnosis this cancer with more precision and accuracy. We aimed to help physicians by computerized models to prognosis of this cancer sooner, without expensive price, and have a less side effect on patients. Therefore, the data collections enter to validation process by *k*-fold method, other process were done, and finally the last model was generated. The data were collected from two physician offices and Javadalaemeh Clinic. Eventually, 208 patients were examined. Evaluation of 64 data mining models was done in Weka and MATLAB software. The evaluation was based on accuracy and precision.

In our study, Bayes-Net with accuracy of 95.67%, precision of 95.70% and sensitivity of 100% was found the best model for prediction and diagnosis of breast cancer. In addition, spread of Ductal is more than Lobular in Kerman.

Advantageous of BN model:

- BN had a high ability for prognosis (42).
- There was absence of access to valuable data sources BN still has a good performance (42).
- It had a high ability in controlling missing data.
- BN had a good ability to deal with unrelated data.

Comparison between ABML and BN models:

- The base of classification used in ABML was random classification (13) but BN was a model that incorporates two kinds of theory (presumption and graphical) to display a relationship between data (43).
- In both of them, percentages of sensitivity were the same, and percentage of accuracy and precision in BN is higher than RBFN.
- ABML had higher sensitivity to data noises, and BN has a good performance to make probability relationships.

Comparison between RF and BN:

- RF was made of some CART (Classification and Regression Trees). These CARTs used some random sample data among the main sample data (12), BN was made of algorithms that can predict with high precision and accuracy.
- RF was user-friendly model because it has just two parameters: The first parameter was number of random trees in forest, and the second parameter was number of predictor variables, which are set into subsets (12). BN had a perfect ability to predict values even in limitation of access to comprehensive data (42).
- In this study, BN model had higher percentages of accuracy, precision, and sensitivity than RF.

Comparison between Bagging and BN:

- Bagging was a model used to produce different models of a predictor (44). BN have algorithms that have many uses such as prognosis.
- Bagging had a considerable accuracy despite turmoil in learning collection it can modify accuracy (44). BN is a great way to represent real conclusions, and it is able to organize real conclusions (43).

## Conclusion

To early predict and undergo prognosis of breast cancer utilization of data mining models is necessary. By a reliable data mining model, we can help physician to early diagnosis of breast cancer. Therefore, the cost of treatments dramatically decreases, and disease progression is prevented.

## Ethical considerations

Ethical issues (Including plagiarism, informed consent, misconduct, data fabrication and/or falsification, double publication and/or submission, redundancy, etc.) have been completely observed by the authors.
